# Gut Symbiont-Driven Adaptive Evolution of Herbivorous Insect–Plant Interactions and Its Ecological Implications

**DOI:** 10.3390/plants15010014

**Published:** 2025-12-19

**Authors:** Junming Li, Yaqi Yu, Lovemore Zulu, Nan Xu, Yanxue Pan, Wenze He, Xunyue Liu, Qiong Rao

**Affiliations:** 1 Zhejiang Key Laboratory of Biology and Ecological Regulation of Crop Pathogens and Insects, College of Advanced Agricultural Sciences, Zhejiang A & F University, Hangzhou 311300, China; 2 College of Plant Protection, China Agricultural University, Beijing 100107, China

**Keywords:** gut symbiont, herbivorous insect, interaction, adaptive evolution, ecological effect

## Abstract

The interaction between plants and phytophagous insects is one of the most complex relationships in ecosystems. By acting as direct third-party participants, gut symbionts redefine this binary antagonistic relationship. This article reviews the roles of gut symbionts in the adaptive evolution of phytophagous insects, highlighting their important roles in degrading plant secondary metabolites, modulating plant defense responses, promoting insect nutrient absorption, and shaping immune phenotypes. Gut symbionts not only enhance the adaptability of insects by degrading plant defense compounds, but also significantly influence their physiological adaptation by manipulating plant defense signaling pathways, regulating the immune system of insects, and promoting their rapid adaptation to external stress. When insects are confronted with environmental changes or shifts of host plants, the dynamic plasticity of the gut symbionts provides them with evolutionary advantages. Reviewing the mechanism of action of intestinal symbiotic bacteria in the adaptive evolution of insects is helpful to deepen our understanding of the ecological interaction process between insects and plants.

## 1. Introduction

Over hundreds of millions of years, the co-evolution between plants and herbivorous insects has created one of the most diverse, complex, and dynamic interaction relationships in terrestrial ecosystems [1]. According to the classic theory proposed by Ehrlich and Raven in 1964, this relationship is described as a prolonged evolutionary “arms race”, in which plants continuously evolve complex physical and chemical defense mechanisms to the pressure from herbivores [2]. And insects have developed adaptive strategies at the levels of morphology, physiology and behavior to overcome these barriers [3,4]. Traditionally, this interaction has been seen as direct antagonism between the plant host and its insect herbivore. However, with the rapid development of molecular ecology, metabolomics and microbiome research, researchers have gradually realized that herbivorous insects are not independent biological entities [5], but rather form highly integrated life complexes with symbiotic microbial communities, especially in the gut [6,7], which are collectively referred to as symbionts. The insect gut symbionts, as a bridge connecting plants and insects [8], are reshaping our understanding of the mechanisms of plant–insect interactions, ecological effects and their evolutionary paths.

The gut symbionts of insects are far from being passive participants in the host’s digestive and metabolic processes, but rather function as “invisible organs” that regulate the host’s physiological metabolism and ecological niche [8,9,10,11]. The functions of these microorganisms are extremely diverse, spanning molecular physiology to community ecology, and they represent a core driving force enabling insects to successfully adapt to diverse phytochemical environments [12]. On the one hand, gut symbionts can directly disrupt the chemical defense systems of plants [13,14]. They deconstruct polysaccharide polymers such as plant cell walls by producing a series of highly efficient and specific enzyme systems [15,16]. More importantly, they can efficiently degrade various plant secondary metabolites that act as defensive compounds, even converting them into usable nutrient resources [17,18]. On the other hand, gut symbionts actively intervene in and manipulate the plants’ defense regulatory network. Some symbiotic bacteria can enter plants via insect secretions to modulate the antagonistic relationship between the two core defense signaling pathways of jasmonic acid (JA) and salicylic acid (SA) at the molecular level or disrupt earlier damage perception signals, thereby inhibiting or misleading the plant’s resistance response [19,20]. However, this regulatory effect is not always beneficial to insects; rather, it plays a dual role.

Beyond this direct interaction of attack and defense, they effectively compensate for the nutritional imbalances in plant-based foods and broaden the potential host range of insects by synthesizing essential amino acids and vitamins and performing nitrogen fixation [21,22,23]. Moreover, gut symbionts can regulate the release spectrum of Volatile Organic Compounds (VOCs) in plants. These chemical signals serve as a communication language between plants and the natural enemies of insects [24,25]. The intervention of microorganisms alters these communication signals and manipulates the indirect defense mediated by natural enemies [26,27,28], thereby influencing the host location and foraging behavior of organisms at higher trophic levels, ultimately regulating pest population dynamics. In addition, gut symbionts also engage in a complex dialogue with the host immune system to maintain intestinal barrier homeostasis. In response to external stresses like plant defense compounds, biopesticides, or chemical insecticides, they profoundly influence the host’s survival strategies and resistance evolution by reshaping its immune phenotype [7].

Therefore, in the complex system of plant–insect interaction, gut symbionts play a crucial role, determining the direction and outcome of the interaction [29]. A thorough analysis of their specific roles and molecular mechanisms in mediating the adaptive evolution of insects is essential for fundamentally revealing the inherent logic of co-evolution between plants and insects. This article aims to systematically review the various roles of gut symbionts within plant–herbivore interaction, and elaborate in depth on their core functions and mechanisms in supplementing host nutrition, degrading plant defense compounds, regulating the insect immune system, and intervening with plant defense signaling pathways. It will further explore the plasticity of the symbionts themselves and how this plasticity drives insects to rapidly adapt to new hosts or environmental stresses, aiming to provide a new theoretical framework for insect adaptive evolution and to offer inspiration for the future development of novel green control strategies targeting the insect microbiome.

## 2. Plant Defense Remodeling Mediated by Intestinal Symbiotic Bacteria

**Figure 1:**
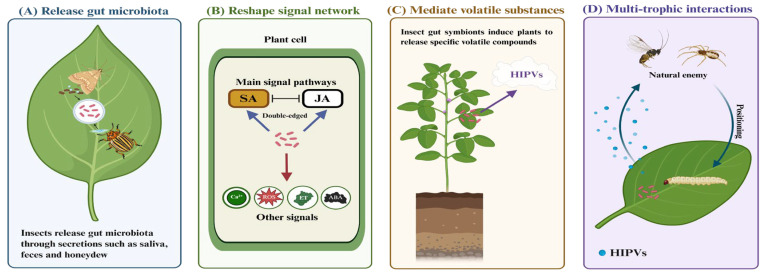
Insect gut symbionts are released through insect behaviors, modulating plant signaling and mediating multitrophic interactions. (**A**) Gut symbionts are released through insect secretions such as saliva, frass, and honeydew, allowing direct contact with plant tissues; (**B**) Gut symbionts modulate plant signaling molecules—including salicylic acid (SA), jasmonic acid (JA), calcium ions (Ca^2+^), reactive oxygen species (ROS), ethylene (ET), and abscisic acid (ABA)—thereby reshaping the plant defense network; (**C**) In addition to altering internal plant signaling, gut symbionts further mediate the emission of herbivore-induced plant volatiles (HIPVs), reshaping external plant signals; (**D**) Gut symbionts released during insect activities remodel both internal and external plant signals, and through HIPVs can attract natural enemies, facilitating multitrophic interactions.

The gut symbionts are the key factors for herbivorous insects to adapt to host plants [30,31]. They are not merely passive residents within the insect, but can actively intervene and reshape the physiological state of plants through insect behaviors such as feeding, secreting and excretion (Figure 1). The core of this regulatory effect lies in their ability to interfere with the complex defense signals of plants, thereby exerting profound ecological effects on both the plants themselves and the multi-trophic interactions within their ecosytems [7,32].

The primary interference targets of gut symbionts are the antagonistic hormone signaling pathways within plants, such as jasmonic acid (JA) and salicylic acid (SA) pathways. Generally, the JA pathway primarily mediates defends against herbivorous insects, while the SA pathway is effective against pathogenic microorganisms (Table 1). The gut symbionts of some insects ingeniously utilize this mechanism [7]. For instance, when the Colorado potato beetle *Leptinotarsa decemlineata* feeds, the symbiotic bacteria in its saliva can induce the potato to activate SA signaling, thereby inhibiting the JA signaling pathway that is central to the plant’s defense against herbivory and ultimately weakening the plant’s defenses against the insect [33]. Similarly, the bacteria in the oral secretions of *Spodoptera litura* can also employ this “diversionary tactic” in the model plant *Arabidopsis*, helping the larvae evade the plant’s immune defense [34]. It is also worth noting that although mites do not belong to the class Insecta, the harm of phytophagous mites to agricultural production is becoming increasingly serious. There are also diverse symbiotic bacterial communities within mites [35]. It can also effectively regulate the JA or SA pathways of plants [36].

The regulatory effect of gut symbionts on plant defense exhibits a significant “dual nature”, with the ultimate direction of this regulation being determined by the unique interaction system formed by the insect, gut microbial strains, and the plant. For example, when a strain of *Enterobacter ludwigii* in the gut of the *Helicoverpa zea* is inoculated onto tomatoes, it can strongly trigger the JA pathway of the plant, thereby enhancing the plant’s resistance to pests [37]. Moreover, the interference range of gut symbionts extends far beyond the JA-SA antagonistic system. They can also affect the early sensing signals of plants such as calciumions (Ca^2+^) and reactive oxygen species (ROS), while regulating multiple hormonal pathways including ethylene (ET) and abscisic acid (ABA). Some gut symbionts can even produce plant hormone analogues, creating a “green island” effect on leaves that delays senescence and provides insects with a more lasting food source [38].

Take Reactive Oxygen Species (ROS) as an example. During the feeding process of insects, intestinal symbiotic bacteria can be transported to the surface of plant wounds along with secretions. The live bacteria and their microbial-associated molecules (MAMPs) therein are perceived by plants, thereby altering the dynamics of reactive oxygen species (ROS) in plants. When plants are normally fed, they will produce a rapid ROS burst through RBOH-like NADPH oxidase, which serves as a signal hub connecting local defense, cell wall reinforcement and JA signaling [39,40]. However, research shows that intestinal symbiotic bacteria can inhibit the accumulation of such ROS, thereby reshaping the plant’s defense system. For example, the *Enterobacter* BC-8 in the intestine of *Leptinotarsa decemlineata* has a “ROS inhibitory effect” on potatoes. The presence of this strain can significantly reduce the accumulation of H_2_O_2_ and phenolic substances in potato leaves, and simultaneously inhibit the activities of POD and protease inhibitor. Simultaneously suppresses POD and protease inhibitor activities, and shifts the defense signaling from JA-mediated anti-herbivore responses toward an SA-biased defense pathway against pathogens [41]. Similar phenomena are also observed in other intestinal bacteria, such as *Acinetobacter* and *Citrobacter* [42].

This remodeling of the internal signals of plants will also further spill over, altering the chemical communication between plants and their surrounding environment. The VOCs released by plants are signals for their “dialogue” with the outside world, especially in attracting natural enemies of pests [43,44,45]. The gut symbionts can also regulate this process. For instance, when the *Nilaparvata lugens* feeds on rice, the gut symbiont in its honeydew act as a strong inducer, prompting rice plants to release increased amounts of attractive volatiles, thereby significantly enhancing the predation efficiency of parasitoid wasps and spiders [46,47]. This regulatory signal can even originate from the excrement of insects; for example, ethyl palmitate produced by the gut symbionts in the feces of *Helicoverpa armigera* and *Spodoptera frugiperda* is itself a chemical signal that can strongly attract parasitic wasps [48].

In summary, the gut symbionts intervene in the physiological processes of plants through the secretions or excretions of insects. The signal-level alterations they trigger modulate plant resistance and the release of volatiles, integrally regulating both plant resistance levels and volatile signal release, ultimately reshaping its role in the multi-trophic food web at the community scale [49].

**Table 1:** Regulation of plant JA and SA signaling pathways by insect gut symbionts.

Insect		Gut Microbiota	Plant	Regulatory Mechanism	Ref.
Order	Species/Family				
Lepidoptera	*Spodoptera frugiperda*	*Klebsiella oxytoca* *Pantoea ananatis* Enterobacteriaceae-1 *Raoultella ornithinolytica*	*Solanum lycopersicum* *Zea mays*	*Raoultella* sp. and *Klebsiella* sp. activate the JA pathway *Pantoea ananatis* and Enterobacteriaceae-1 suppress the JA pathway	[50]
	*Spodoptera litura*	*Staphylococcus epidermidis*	*Arabidopsis thaliana*	Activate the SA pathway and suppress the JA pathway	[51]
	*Helicoverpa zea*	*Enterobacter ludwigii*	*Solanum lycopersicum*	Activate the JA pathway and suppress the SA pathway	[37]
	*Plutella xylostella*	*Bacillus cereus* *Micrococcus* sp. *Enterobacter* spp. *Staphylococcus haemolyticus*	*Arabidopsis thaliana*	Suppress the JA and SA pathway	[52]
	*Helicoverpa armiger*	Not specifically identified	*Arabidopsis thaliana* *Gossypium hirsutum*	Suppress the JA pathway	[34]
Hemiptera	*Sitobion miscanthi*	*Hamiltonella defensa*	*Triticum aestivum*	Suppress the JA and SA pathway	[53]
	*Bemisia tabaci*	*Hamiltonella defensa*	*Solanum lycopersicum*	Suppress the JA pathway	[54]
	*Nilaparvata lugens*	*Acinetobacter soli* *Serratia marcescens* *Staphylococcus sciuri*	*Oryza sativa*	Activate the JA pathway	[46]
	*Nezara viridula*	*Serratia* *Pantoea* *Sodalis* *Yeast*	*Brassica nigra* *Solanum nigrum* *Securigera varia* *Glycine max* *Brassica juncea* *Helianthus annuus*	Suppress the JA and SA pathway	[19]
Diptera	*Bactrocera dorsalis*	*Providencia* *Klebsiella*	Fruits such as citrus fruits and mangoes	Activate the JA and SA pathway	[55]
Coleoptera	*Leptinotarsa decemlineata*	*Pseudomonas* *Enterobacter* *Stenotrophomonas*	*Solanum lycopersicum* *S. melongena* *S. dulcamara* *S. caroliense* *S. tuberosum* *S. rostratum*	Activate the SA pathway and suppress the JA pathway	[20,56]

## 3. The Intestinal Microbiota in the Degradation of Phytochemicals

**Figure 2:**
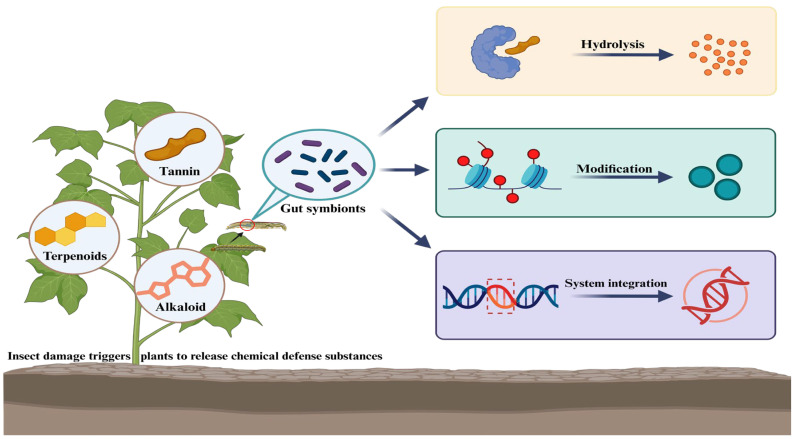
Insect gut symbionts degrade plant defensive compounds through three hierarchical mechanisms to enhance ecological adaptability. Faced with herbivore feeding stress, plants have evolved tannins, alkaloids, terpenoids, and other compounds that impair insect digestion. In response, insect gut symbionts counteract these plant chemical defenses through hydrolysis, chemical modification, and even the evolution of clustered metabolic modules.

Through long-term co-evolution with insects, plants have developed a chemical defense system based on compounds such as tannins, alkaloids, terpenoids, and resin acids, which can suppress insect digestion, impair development, and reduce reproduction [57,58,59]. However, insects do not rely solely on their own detoxification enzyme to overcome these defenses. Their intestinal symbiotic bacteria alleviate the toxicological stress brought by phytochemicals through targeted enzymatic transformation and metabolic reprogramming [60,61]. This cooperative detoxification process involves the following complementary mechanisms (Figure 2).

### 3.1. Hydrolysis

Certain gut symbionts employ hydrolytic enzymes, such as tannase and phenolic esterase to degrade high-molecular secondary metabolites into small-molecule products. This not only reduces their interference with host protein activity and intestinal function but also lowers oxidative stress, thereby alleviating the toxic pressure of plant defense chemicals. For example, *Bacillus pumilu* isolated from the gut of the *Acrobasis nuxvorella* exhibits a strong tannase activity, which can convert the hydrolyzable tannin in pecans into gallic acid and glucose, thereby alleviating the toxicity of tannin on the host and maintaining its normal growth [62]. Similarly, *Bacillus* and *Acinetobacter* isolated from the gut of the oak leaf-feeding *Nothomyllocerus illitus* can degrade tannin, thereby enhancing the feeding, growth and reproductive performance of the larvae [63]. A recent study also found that among the gut symbionts of *Monochamus saltuarius*, the genus *Pseudomonas* is one of the most closely associated with various metabolites including phenolic acids, lignin, and toxic defense compounds; furthermore, genera such as *Acinetobacter* and *Enterobacter* also appear to participate in this process. At the phylum level, Proteobacteria and Firmicutes were found to be the most abundant [64].

### 3.2. Chemical Modification

Gut bacteria can reduce the interference of plant toxins with the nervous, endocrine, and immune systems through chemical modification reactions such as demethylation, reduction/hydroxylation, and structural transformation. For example, gut bacteria such as *Pseudomonas* in the *Hypothenemus hampei* express enzymes related to caffeine N-demethylation (*ndm* gene cluster), which can convert caffeine into low-toxicity derivatives and significantly reduce its toxicity [31]. In the *Curculio chinensis*, *Acinetobacter* sp. AS23 reduces the toxicity of the plant toxin tea saponins (TS) by degrading it within the gut, enabling the insect to adapt to a fruit environment with higher concentrations of tea saponins [65]. AS23 decomposes the benzene ring structure and glycosidic bonds of TS through pathways including styrene degradation, phenylpropane metabolism, and phenylacid degradation, converting them into low-toxicity compounds, thereby significantly reducing the negative impact of tea saponins on the host. Furthermore, in the study of *Psylliodes chrysocephala*, researchers found that *Pantoea* strains were isolated and identified in its gut microbiota. After inhibiting the microbiota, unmetabolized isothiocyanates (ITCs) accumulated in the insects’ bodies. However, when these strains were replenished, the degradation of ITCs was restored, directly demonstrating the involvement of symbiotic bacteria in the detoxification of isothiocyanates in this insect [66]. Similarly, in the diamondback moth (*Plutella xylostella*), the gut bacterium *Enterobacter* sp. EbPXG5 degrades the flavonoid secondary metabolite kaempferol both in vivo and in vitro. Germ-free insects showed significant developmental impairments when exposed to kaempferol, but supplementation with EbPXG5 restored growth, highlighting the critical role of microbial detoxification in host adaptation [67].

### 3.3. System Integration

In addition to the single enzymatic detoxification, some symbiotic bacteria have further evolved metabolic modules clusters that can systematically break down complex defense compounds. These bacteria carry specialized metabolic gene clusters capable of systematically degrading complex and highly defensive liposoluble compounds, such as terpene resin acids, thereby enhancing the adaptability of insects exposed to these plant defense substances [68]. For instance, a “dit” gene cluster was discovered in the gut microbiota of *Hylobius abietis*, which can efficiently degrade diterpene resin acids from *Picea abies*. Upon elimination of the microbiota via antibiotic treatment, the degradation capacity significantly decreases, accompanied by synchronous decreases in egg production and hatchability; these traits recover after the microbiota is restored [68]. A recent study on *Hyphantria cunea* revealed that when its southern population feeds on the newly encountered host plant *Metasequoia glyptostroboides*, the abundance of certain gut symbionts, including *Bacteroides*, *Blautia* and *Coprococcus*, is closely related to the larvae survival rate on this host. Microbial transplantation experiments further demonstrated that these microbial communities enhance the survival performance of the northern population on *Metasequoia*. These results indicate that such bacterial genera may provide adaptive advantages to the host against plant chemical defenses through yet unconfirmed metabolic pathways [69].

## 4. Gut Microbiota Regulate the Immune Strategies of Insects

Gut symbionts act as crucial mediators linking plant defense and insect immune responses. The defense stress induced by plants can damage the intestinal barrier, promote bacterial penetration into the epithelium, and activate the host immune system, thereby synergistically enhancing the stress effect [70]. In the interaction between *Spodoptera frugiperda* and corn, cystease and chitinase expressed by corn can disrupt the (peritrophic matrix of the midgut, allowing bacterial species such as *Enterobacter* and *Klebsiella*, which are normally confined in the intestinal lumen, to cross the gut wall and enter the hemocoel. This triggers systemic bacterial translocation and activation of humoral immunity, manifested as typical reactions such as increased phenol oxidase activity and elevated hemocyte count [71]. Similar phenomena have been verified in the *Bombyx mori*: antibiotic treatment significantly reduced the gut microbiota abundance, inducing phenotypic alterations such as a thinner, structurally looser, and more permeable peritrophic matrix, while concurrently repressing the expression of key genes related to antimicrobial peptides and the peritrophic. These effects collectively exacerbated the impact of plant immune stress [72].

Meanwhile, some bacterial strains help maintain community homeostasis and establish a “chemical immune barrier” by regulating immune pathways such as Toll, IMD, and Duox-ROS, and by secreting bacteriocins. The diet of *S. frugiperda*, derived from different plant species, significantly shapes the composition and functional potential of its midgut microbial communities [73]. Antibiotic intervention suppresses the Toll/IMD pathway, whereas reintroduction of *Enterococcus mundtii* or *E. gallinarum* enhances resistance to the baculovirus AcMNPV. Moreover, the *S. frugiperda* can also attract the corn endophytic bacterium *Pantoea dispersa* to colonize its gut, which effectively metabolizes the plant defense substances such as benzodiazines (BXs) [74]. This colonization enhances growth capacity while simultaneously lowering the activation threshold of the Duox-ROS axis. In *Spodoptera littoralis*, the resident *E. mundtii* secretes the class IIa bactericin mundticin KS, which selectively suppressing pathogenic bacteria to maintain intestinal homeostasis, thereby forming a defensive line similar to a “chemical immune barrier” [75]. This process synergistically enhances the host’s physiological adaptability and immune resilience of the host under plant defense pressure.

**Figure 3:**
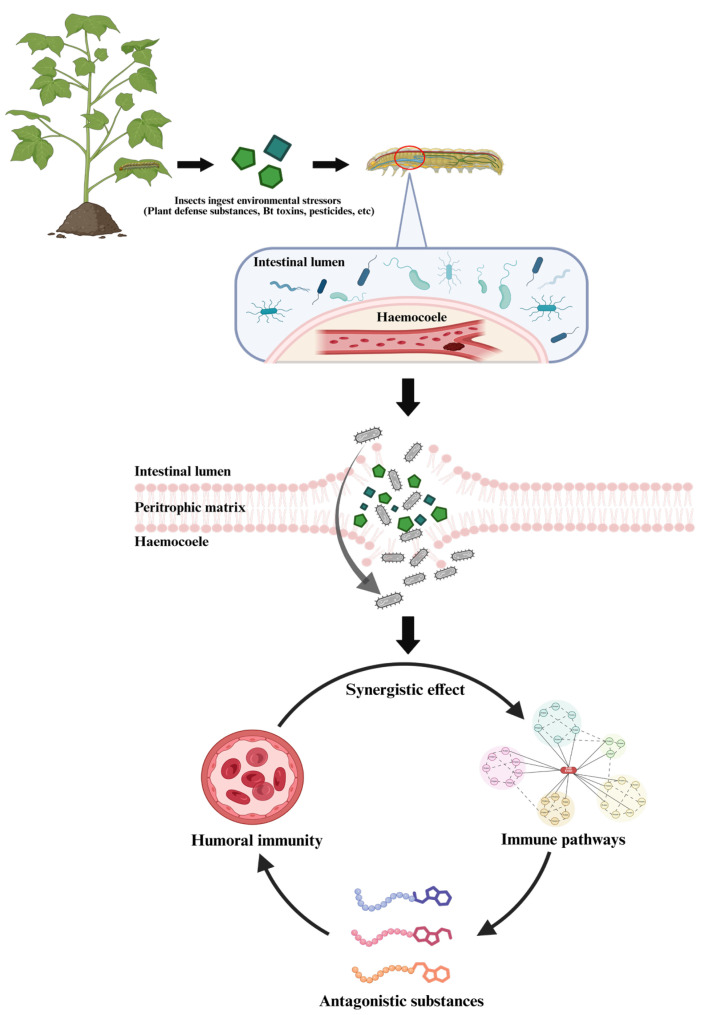
Environmental stressors disrupt the insect gut barrier, enabling bacterial translocation and triggering host immune and physiological responses. Insects ingest plant defensive compounds, toxins, and pesticides during feeding, and these stressors can disrupt the gut barrier and amplify physiological damage. In response, gut symbionts cooperate with the host by modulating humoral immunity, regulating immune signaling pathways, and secreting antagonistic substances to support insect immune defenses.

In the context of *Bt* crop applications, studies further indicate that gut symbionts can reshape the insect innate immunity and promote bacterial translocation following midgut epithelial damage induced by bacteriocins, thereby altering host susceptibility to *Bt* toxins and pesticides [76,77]. Taking *Plutella xylostella* as an example, the Cry1Ac toxin induces the Toll-like receptor/IMD pathway and upregulates antimicrobial peptides in a manner that depends on the endogenous gut microbiota. Depleting the gut microbiota reduces the host’s susceptibility to Cry1Ac, whereas re-inoculation with *Enterococcus mundtii*, *Carnobacterium maltaromaticum*, and *Acinetobacter guillouiae* isolated from the cabbage looper gut restores sensitivity. This demonstrates that gut microbiota–host immune interactions amplify the effects of *Bt* toxins [78,79]. Furthermore, the extracellular polysaccharides derived from *Bt* can also interact with Cry1Ac to enhance the sensitivity of sterile *Plutella xylostella* larvae to the toxins, indicating that microbial metabolites can also intervene and amplify this interaction effect. Similar mechanisms have also been found in other lepidopterans, though their dependence on microbiota varies systematically. In some host–microbiota systems, the microbiota is not essential for toxicity [80,81,82]. Regarding chemical control, *Enterococcus* with *P. xylostella*’s gut can also enhance the host’s tolerance to pyrethroid insecticides [83]. A systematic reprogramming of the antimicrobial peptide transcriptional profile reveals that the underlying mechanism does not rely exclusively on direct pesticide degradation by microorganisms, but rather operates through an “immunodetoxification coupling” process that indirectly enhances tolerance. In summary, gut symbionts regulate the host’s immune response and physiological adaptation to diverse stressors, including plant defenses, biocontrol agents, and chemical pesticides. This regulation is achieved through multiple mechanisms, such as maintaining intestinal barrier homeostasis, regulating immune pathways, secreting antagonistic metabolites, and combining with immunophenotypic remodeling (Figure 3). Thus, gut symbionts are not merely an ecological companion of insects, but a core driving factor that actively shapes their immune ecology and adaptive evolution of insects [84,85].

## 5. Digestive Enhancement and Nutrient Synthesis Mediated by Gut Microbiota

Insects frequently depend on nutritionally unbalanced food sources, a challenge especially pronounced in sap-feeding species (Table 2). These insects must cope with plant phloem sap that is high in sugars but lacking essential amino acids and other nutrients. In this process of nutritional adaptation, the gut microbiota plays a key role [86,87].

**Table 2:** Multiple insect gut symbionts assist in the degradation of cell wall components.

Insect		Food	Gut Microbiota	Enzyme Provided	Ref.
Order	Species/Family				
Lepidoptera	*Helicoverpa armigera*	Leaves, buds and fruits, etc.	*Bacillus* *Klebsiella*	Cellulases Hemicellulose	[88]
	*Bombyx mori*	Mulberry leaves	*Aeromonas* *Bacillus circulans* *Citrobacter freundii* *Escherichia coli* *Enterobacter* *Erwinia* *Klebsiella pneumoniae* *K. pneumoniae* *Proteus vulgaris* *Pseudomonas fluorescens* *P. aeruginosa* *P. vulgaris* *Serratia liquefaciens*	Cellulases Hemicellulose Pectinases	[89]
	*Cossus cossus*	Xylem	*Bacillus circulans*	Cellulases	[90]
Coleoptera	*Cassida rubiginosa*	Leaves	*Candidatus* Stammera capleta	Pectinases	[16,91,92]
	*Protaetia brevitarsis*	Decaying organic matter	*Bacteroidetes* *Firmicutes*	Cellulases Hemicellulases	[93]
	*Lepidiota mansueta*	Root	*Bacillus* *Klebsiella* *Providencia*	Cellulases	[94]
	*Anoplophora glabripennis*	Xylem	*Brachybacterium* *Corynebacterium* *Enterococcus* *Fusarium solani* *Gibbsiella* *Pseudomonas* *Sphingomonas* *Wolbachia* *Xanthomonas*	Cellulases Hemicellulases Ligninase	[95,96]
Hemiptera	*Pyrrhocoris apterus* *Dysdercus fasciatus*	Seed	Actinobacteria	Hemicellulases	[97]
	*Nezara viridula*	Tissue juice	*Commensalibacter* *Sodalis*	Pectinases Hemicellulases	[19]
Diptera	*Bactrocera dorsalis*	Tissue juice	PSG1 PSG3 (Not specifically identified)	Cellulase Xylanase Pectinase	[98]
	*Hermetia illucens*	Decaying tissue	Proteobacteria Firmicutes Actinobacteria Bacteroidetes	Cellulases Ligninases	[99]
Hymenoptera	*Sirex noctilio*	Xylem	Acinetobacter Bradyrhizobium Burkholderia Nitrosospira Pseudomonas Ralstonia Ruminobacter Streptomyces Trichoderma reesei Zoogloea γ-Proteobacteria	Cellulases Hemicellulases Pectinases Ligninase	[100,101,102]
	*Apis mellifera*	Pollen	*Bifidobacterium asteroids* *Gilliamella apicola*	Pectinases	[15]
Blattaria	Termite	Xylem	Actinomycetota Acidobacteriota Bacillota Bacteroidota Fibrobacterota Pseudomonadota Spirochaetota	Cellulases Pectinases Ligninase	[103,104]

Insects commonly encounter the challenge of tough and indigestible cell wall components when feeding on plants. To overcome this, intestinal symbiotic bacteria can produce hydrolases that are absent in the host insects [105], thereby substantially improving digestion and utilization of plant polymers. While most insects lack enzymes such as cellulase, pectinase, and ligninase, but examples of these enzymes being provided by symbiotic bacteria have been found in various lepidoptera, coleoptera, blattodea, and hymenoptera. For instance, actinomycetes and panomycetes have been identified in the gut of *Sirex noctilio*, which can secrete cellulase to assist the host in decomposing xylem cellulose [96,100,101]. Moreover, cellulose and hemicellulose fibers within plant cell walls are embedded in the pectin matrix and require initial degradation by pectinase to expose the cellulose for further decomposition. Studies have found that insect gut bacteria are indeed capable of producing pectinase. For example, the symbiotic bacterium *Stammera* of the *Cassida rubiginosa* carries the necessary genes for pectin degradation [16,92,106]. In addition, lignin is a more complex and difficult-to-decompose polymer in plants. Although its decomposition mainly relies on symbiotic fungi. Nevertheless, bacterial strains capable of lignin degradation have been isolated from the guts of insects such as termites, scarab beetles, and longhorn beetles [93,96,103]. The phenomenon of digestive symbiosis is evident in these examples, where gut bacteria provide insects with a rich repertoire of hydrolytic enzymes, thereby allowing them to break down and utilize plant material that would otherwise be indigestible.

Beyond facilitating the digestion of structural carbon sources, intestinal symbiotic bacteria also play a significant role in compensating for nutritional deficiencies in the diets of herbivorous insects. Many plant tissues are nutritionally unbalanced, commonly characterized by limitations in nitrogen, essential amino acids, and vitamins. Insects have evolved various strategies mediated by intestinal bacteria, to alleviate nutritional constraints [107]. Nitrogen-fixing intestinal symbiotic bacteria have been identified in certain coleoptera and diptera insects, enabling them to convert atmospheric nitrogen fixation into biologically usable forms [108]. For instance, symbiotic bacteria exhibiting nitrogenase activity have been identified in the larvae of the *Odontotaenius disjunctus* and the *Ceratitis capitata*, both of which can create additional nitrogen sources for their hosts [85,109]. Similarly, the gut microbiota of *Bactrocera dorsalis* facilitates the recycling of nitrogen- compounds from the host’s metabolic waste (with the assistance of bacteria such as *Morganella morganii* and *Klebsiella oxytoca*) through the nitrogen waste reuse pathway and synthesizes essential amino acids for the host to absorb [110]. Furthermore, herbivorous insects often obtain insufficient vitamins from their host plants. For example, symbiotic bacteria in the midgut of *Riptortus pedestris* synthesize B vitamins, thereby compensating for the vitamin deficiency in legume seeds [111]. Similarly, some studies have found through genomic analysis that certain herbivorous ants (e.g., species of the genus *Dolichoderus*) carry essential amino acid and multiple vitamin synthesis pathways in their symbiotic Bartonellaceae family [112]. It is evident that intestinal symbiotic bacteria have significantly broadened the available nutrient sources for herbivorous insects through nitrogen fixation, nitrogen recycling and nutrient synthesis.

## 6. The Plasticity of the Gut Microbiota and the Rapid Adaptation of Insects

**Figure 4:**
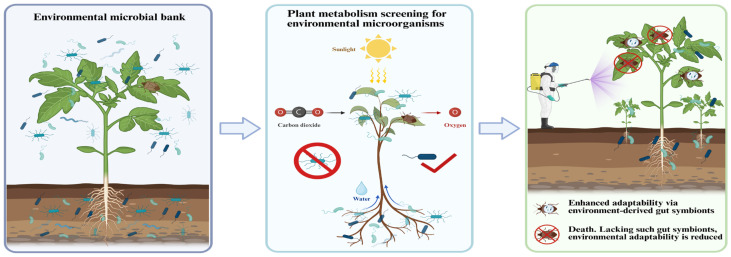
Plant-selected environmental microbes increase the opportunities for gut symbiont acquisition, enhancing insect ecological adaptability. Many gut symbionts are not solely obtained through maternal vertical transmission but are continuously acquired from the environment, which functions as a microbial bank. Plants selectively filter specific microbial taxa through their metabolic processes, and insects, by feeding on these plants, rapidly acquire these plant-selected microbial communities and the functional traits they confer. In the middle panel, the red √ indicates successful microbial selection.

In the long-term competition between herbivorous insects and plants, the core driving force of insects stems not only from the evolution of their own genomes but also from a symbiotic complex characterized by high plasticity jointly formed by the insects and their intestinal microorganisms. The composition and function of this complex are not static, but can be dynamically adjusted and reconstructed according to external environment, especially plant chemical signals, enabling rapid adaptation [84,113]. This plasticity lies in the “environmental acquisition” and “ecological filtering” mechanisms of microbiota [114]. The gut microbiota of many insects is not strictly passed vertically from the mother but is continuously obtained from the environment. Plant microhabitats such as the leaf surface and rhizosphere soil constitute a resource pool that insects can utilize [115]. However, insects do not passively acquire all environmental microbes. Plants act as “ecological filters” through secondary metabolites and nutrients, screening out specific microorganisms that can survive and function [116]. Therefore, the insect gut microbiota constitutes a dynamic system that is precisely regulated under the dual effects of environmental supply and selection. This dynamic acquisition and screening mechanism endows insects with the rapid adaptability to “leverage external forces”, and even enables them to achieve “phenotypic leaps”. This is fully reflected in the interaction system between the *Riptortus pedestris* and its intestinal symbiotic bacteria *Burkholderia* (Figure 4). The *Burkholderia* in the gut of the *Riptortus pedestris* is not inherited but is re-obtained from the rhizosphere of the soil in each generation and established in the intestinal glandular fossa [117]. This model brings about astonishing adaptability: when organophosphorus pesticides are applied to farmland for a long time, *Burkholderia* strains harboring pesticide-detoxification genes accumulate in the soil. By establishing symbiosis with these pre-adapted strains, the bean stink bug can rapidly acquire high tolerance to pesticides, a rapid adaptation that traditional evolution cannot achieve [118]. This microbe-mediated phenotypic transition has also been observed in other stink bugs within the same family [119].

The plasticity of the gut microbiota is a key factor enabling insects to expand their dietary range and further increase their geographical distribution. When insects are confronted with new host plants, their intestinal microbiota can often reorganize rapidly to adapt to the new plant environment. For example, within just a few generations after the host change, the structure of *Bactrocera cucurbitae*’s gut microbiota and metabolic function will undergo significant adjustments, thereby adapting to the chemical composition of the new host [120]. Similarly, research on *Hyphantria cunea* has further revealed the significance of this plasticity for geographic range expansion. The southern population exhibits strong adaptability when facing the originally unsuitable host *Metasequoia glyptostroboides*. When the gut microbiota from this southern population was transplanted into individuals from the northern population, the survival rate on this host increased significantly. This indicates that the gut microbiota not only helps insects overcome chemical defense barriers but also serves as a key factor in enabling them to transcend geographical boundaries and achieve range expansion [69].

Long-term selection pressure turns flexible microbial partnerships into core functional modules. Even though microbiota change easily, constant stress leads to the same key bacteria being selected. In conifer forests, bark beetles (such as *Ips* and *Dendroctonus*) consistently host core bacteria (like *Pseudomonas*, *Serratia*, and *Rahnella*) in their guts. These bacteria efficiently degrade wood fibers, metabolize toxic terpene resins and replenish nitrogen nutrients [121]. This cross-species and cross-regional functional unit can be regarded as an efficient adaptive module that has been stabilized from a dynamic symbiotic relationship under long-term co-evolutionary pressure [122].

## 7. Concluding Remarks and Future Perspectives

**Figure 5:**
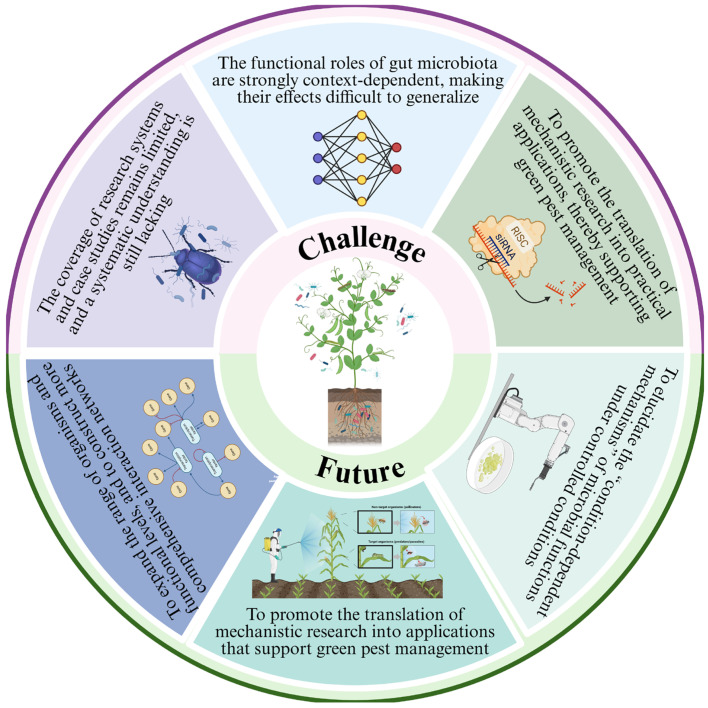
Challenges and prospects of plant–gut symbiont–insect interactions.

This review systematically summarizes the multi-level roles of gut symbionts in mediating herbivorous insect–plant interaction, including intervention in interfering with plant defense signals, degradation of plant secondary metabolites, regulation of host immune status, compensation for nutritional limitations, and promotion of rapid adaptation of insects during host transition and environmental changes. These functions have all been observed in the actual insect–plant interaction system (Figure 5).

However, existing evidence indicates that the function of the gut microbiota is not fixed but is significantly influenced by the phytochemical background, the nutritional status of the host, and fluctuations in the composition of the microbiota. For instance, some strains can weaken the defense of host plants, but in other systems, the same strains can enhance the plant’s defense [123]. For instance, some bacterial colonies can significantly enhance the nutrient absorption efficiency of insects, but under the condition of relatively nutrient-rich food, this compensatory effect is not obvious [124,125].

Furthermore, some microbiota-mediated factors with potential ecological significance have still not received sufficient attention. Take plant protease inhibitors (PIs) as an example. PIs are important defense factors for plants against herbivorous insects. For a long time, the response mechanism of insects to PIs has mostly been regarded as relying on endogenous digestive physiological regulation [126]. But existing studies have shown that some intestinal symbiotic bacteria of insects can participate in regulating the activity of plant PIs [127,128]. However, there is currently a lack of clear evidence regarding the direct synthesis or inhibition of protease inhibitors (PIs) by insect intestinal symbiotic bacteria. But in insect pathogenic bacteria such as *Photorhabdus luminescens* and *Xenorhabdus bovienii*, multiple protease inhibitors that can affect insect fitness have been identified [129,130,131]. This implies that similar mechanisms may also exist in the gut microbiota, but have not yet been systematically revealed.

Although existing studies have fully demonstrated that intestinal symbiotic bacteria play multi-level roles in the interaction between herbivorous insects and plants, their influence effects still have considerable dynamics and systematic complexity. Given the current amount of available literature and the concentration of research systems, reports on some insect groups, symbiotic strains and their metabolic factors are relatively limited. There may be situations where case coverage is insufficient or relevant evidence has not been included. These factors may lead to an incomplete understanding of the role played by the gut microbiota in the interaction between herbivorous insects and plants. Future research can focus on further analyzing the conditional mechanisms of microbiota action under controllable experimental conditions and clarifying how dynamic symbiotic relationships affect the direction of microbiota action. In addition, it is still necessary to further verify the mechanism of action of other regulatory factors in the interaction system in order to provide inspiration for green prevention and control technology mediated by symbiotic bacteria.

## Data Availability

No new data were created or analyzed in this study. Data sharing is not applicable to this article.
